# mRNAs containing NMD-competent premature termination codons are stabilized and translated under UPF1 depletion

**DOI:** 10.1038/s41598-017-16177-9

**Published:** 2017-11-20

**Authors:** Won Kyu Kim, SeongJu Yun, Yujin Kwon, Kwon Tae You, Nara Shin, Jiyoon Kim, Hoguen Kim

**Affiliations:** 1 Department of Pathology and Brain Korea 21 PLUS Projects for Medical Science, Yonsei University College of Medicine, Seoul, 03722 Republic of Korea; 2 Broad Institute of MIT and Harvard, Cambridge, MA 02142 USA; 3 Department of Genetics, Harvard Medical School, 77 Avenue Louis Pasteur, Boston, MA 02115 USA; 4 Department of Pharmacology and Brain Korea 21 PLUS Projects for Medical Sciences, Yonsei University College of Medicine, Seoul, 03722 Republic of Korea

## Abstract

mRNAs containing premature termination codons (PTCs) are rapidly degraded through nonsense-mediated mRNA decay (NMD). However, some PTC-containing mRNAs evade NMD, and might generate mutant proteins responsible for various diseases, including cancers. Using PTC-containing human genomic β-globin constructs, we show that a fraction (~30%) of PTC-containing mRNAs expressed from NMD-competent PTC-containing constructs were as stable as their PTC-free counterparts in a steady state. These PTC-containing mRNAs were monosome-enriched and rarely contributed to expression of mutant proteins. Expression of trace amounts of mutant proteins from NMD-competent PTC-containing constructs was not affected by inhibition of eIF4E-dependent translation and such expression was dependent on a continuous influx of newly synthesized PTC-containing mRNAs, indicating that truncated mutant proteins originated primarily in the pioneer round of translation. The generation of mutant proteins was promoted by UPF1 depletion, which induced polysome association of PTC-containing mRNAs, increased eIF4E-bound PTC-containing mRNA levels, and subsequent eIF4E-dependent translation. Our findings suggest that PTC-containing mRNAs are potent and regulatable sources of mutant protein generation.

## Introduction

Nonsense-mediated mRNA decay (NMD) is a quality-control mechanism at the level of translation that degrades PTC-containing mRNAs generated by nonsense/frameshift mutations, gene rearrangement, or splicing^1–3^. If translated, PTC-containing mRNAs have the potential to produce deleterious truncated proteins that could derange cellular function through gain-of-function or dominant-negative activity. Central to the NMD pathway, various factors, such as exon-junction complexes (EJCs) and UPF complexes, play key roles^4,5^. The ultimate goal of NMD is to prevent the generation of truncated mutant proteins by degrading the PTC-containing mRNAs.

If mutant mRNAs contain a PTC in the last exon, they are not efficiently recognized by NMD (NMD-irrelevant), and truncated mutant proteins are expected to be generated from these mRNAs in human cells^6^. We previously reported that NMD-irrelevant PTC-containing mRNAs generated by frameshift mutations in the last exon were intact and translated to truncated mutant proteins with neopeptides (neoantigen) in colorectal cancers with high microsatellite instability (MSI-H). These truncated mutant proteins are rapidly removed in human cells by proteasome-mediated degradation, which is another check point, and can be used as potent tumor antigens^7^. Except for the mutant mRNAs containing a PTC in the last exon, the other mutant mRNAs containing PTC are expected to be degraded by NMD. However, studies have provided growing evidence that some NMD-competent PTC-containing mRNAs evade NMD, exist stably in human cells, and/or can be rescued from NMD surveillance under specific physiological conditions^8–12^. It is crucial to determine whether mutant proteins are generated from these PTC-containing mRNAs that evade or are rescued from NMD, because generation of mutant proteins is directly linked to diseases, especially cancers^6,13,14^. Moreover, recent studies have shown that mutational load and neoantigen load are significantly associated with clinical benefits when immunotherapies are applied to patients with melanoma or MSI-H colorectal cancers. This finding highlights the importance of gaining a deeper understanding of mechanisms underlying mutant protein generation^15,16^.

Using general and Tet-Off expression constructs containing human genomic β-globin, we show that some PTC-containing mRNAs from NMD-competent PTC-containing constructs were stably expressed in a steady state. These mRNAs were mostly associated with monosomes and rarely contributed to continuous mutant protein generation. Trace amounts of mutant proteins were detectable from NMD-competent PTC-containing constructs, and they were expressed primarily during the pioneer round of translation of newly synthesized PTC-containing mRNAs. We also provide evidence that NMD inhibition by UPF1 or SMG1 knockdown induced bulk production of mutant proteins through eIF4E-dependent translation. Overall, our findings indicate that trace amounts of truncated mutant proteins are constantly generated in the pioneer round of translation and that generation of mutant proteins can be significantly enhanced through eIF4E-dependent translation of PTC-containing mRNAs by inhibiting UPF1 or SMG1.

## Materials and Methods

### Cell lines and reagents

HeLa and HEK293 cells were obtained from American Type Culture Collection (ATCC, Manassas, VA, USA). Cells were maintained in Dulbecco’s Modified Eagle Medium (DMEM) containing 10% fetal bovine serum (Life Technologies, Carlsbad, CA, USA) according to ATCC guidelines. Cells were treated with proteasome inhibitor MG132 (Merck, Kenilworth, NJ, USA) and 4EGi-1 (Santa Cruz Biotechnology, TX, USA), which inhibits the interaction of the translation initiation factors eIF4E and eIF4G, to specifically block eIF4E-dependent translation^17^. To confirm the stability of mRNAs expressed from β-globin constructs, cells were treated with Actinomycin D (ActD; Sigma, St. Louis, MO, USA) to inhibit transcription.

### Semi-qPCR and qPCR

Total RNA was isolated from cells transfected with expression constructs encoding β-globin and EGFP using illustra RNAspin Mini Kits (GE Healthcare, Chalfont St. Giles, UK), and RT was carried out using 2 μg of RNA. qPCR was conducted using the ABI PRISM 7500 Sequence Detector (Applied Biosystems, Foster City, CA, USA) and SYBR Premix Ex TaqII (TaKaRa, Shiga, Japan), according to the manufacturers’ guidelines. β-globin mRNA levels were normalized to that of EGFP.

### Plasmid construction and transfection

Two human β-globin expression constructs were prepared, one containing genomic DNA, including introns, and the other containing cDNA. To generate the genomic DNA expression vector (gBglo-WT), the three exons of the *β-globin* gene and intervening introns were cloned into vector PCMV10 containing a 3xFLAG tag. Point mutagenesis was carried out to generate mutant constructs gBglo-P39 and gBglo-P66 (PTC located in exon 2) and gBglo-P101 and gBglo-P127 (PTC located in exon 3, the last exon). To generate the cDNA expression vector (cBglo-WT), coding regions of the *β-globin* gene were amplified by PCR from cDNA obtained from RNA isolated from cells transfected with gBglo-WT and cloned into vector PCMV10 containing a 3xFLAG tag. Using cBglo-WT, mutant constructs cBglo-P39, cBglo-P66, cBlgo-P101 and cBglo-127 were generated by point mutagenesis. To construct Tet-Off genomic β-globin expression vectors, the region of genomic *β-globin* in the PCMV10 vector with a 3xFLAG was cleaved and subcloned into the pTet-Off vector (Clontech, Mountain View, CA, USA). Cells transfected with pTet-Off β-globin expression vectors were treated with doxycycline (Dox; Clontech) to repress transcription. The coding regions of 4E-BP1 and eIF4E were cloned into PCMV10 containing a HA tag. Cells were cotransfected with vector CMV10-EGFP as internal control. Cells were transfected with each β-globin expression construct alone and together with short interfering RNAs (siRNAs) targeting EIF4Alll, Y14, UPF1, UPF2, MAGOH, or SMG1 (Bioneer, Daejeon Korea) using Lipofectamine 3000 (Life Technologies) according to the manufacturer’s protocol and harvested 3 days later. The primers used for cloning and nucleotides targeted by siRNAs are shown in Supplementary Table S1.

### Western blotting

Total proteins were prepared from transfected cells using Passive Lysis Buffer (Promega, WI, USA), and 30 μg of each sample were separated by SDS-PAGE and transferred to PVDF membranes. After blocking with Tris-buffered saline with Tween 20 (TBST) containing 5% skim milk, blots were incubated for 1 h at room temperature with primary antibodies against GAPDH (Trevigen, MD, USA), HA (Santa Cruz Biotechnology), UPF1 (Cell Signaling Technology, MA, USA), SMG-1 (Cell Signaling Technology), Y14 (Santa Cruz Biotechnology), UPF2 (Santa Cruz Biotechnology), EIF4Alll (Proteintech, Manchester, UK), MAGOH (Santa Cruz Biotechnology), FLAG (Sigma), GFP (BD Biosciences, NJ, USA), eIF4E (Santa Cruz Biotechnology), and HIF1-α (Cell Signaling Technology). Horseradish peroxidase (HRP)-conjugated secondary antibody (Santa Cruz Biotechnology) was used.

### Polysome fractionation

Forty-eight hours after transfection of expression vectors, HeLa cells were incubated with 100 µg/mL cycloheximide for 5 min at room temperature and washed three times with ice-cold PBS. Cells were collected by scraping into PBS and then incubated in lysis buffer (15 mM Tris-HCl (pH 7.4), 3 mM MgCl_2_, 10 mM NaCl, 0.5% Triton X-100, 100 μg/mL cycloheximide, and 200 U RNasin). Nuclei and debris were removed by centrifugation at 12,000 *g* for 2 min. One milliliter of each sample was layered onto an 11-mL 10–50% sucrose gradient and centrifuged for 2 h at 4 °C in an SW41 rotor at 39,000 rpm. Twelve fractions were collected from the top of each gradient, with concomitant measurements of absorbance at 254 nm, using a fraction collection system. RNA was extracted from each fraction using TRIZOL reagent (Life Technologies) and analyzed by Semi-qPCR.

### Immunoprecipitation and m^7^GTP Sepharose pull-down assay

For the immunoprecipitation assay, HeLa cells transfected with HA-4E-BP1 or treated with 4EGi-1 were lysed with Passive Lysis Buffer and lysates were incubated overnight with eIF4G antibody (Cell Signaling Technology). After the incubation of lysates with A/G PLUS Agarose (Santa Cruz Biotechnology), beads were washed 5 times with Tris-buffered saline (TBS) and resuspended with 3 × SDS sample buffer. For the m^7^GTP Sepharose pull-down assay, cell lysates were incubated with m^7^GTP Sepharose (Jena Bioscience, Jena, Germany) for 2 h at 4 °C and Sepharose beads were washed 5 times with NT2 buffer (50 mM Tris-HCl (pH 7.4), 150 mM NaCl, 1 mM MgCl_2_, 0.05% NP40). Then, Sepharose beads were resuspended with 3 × SDS sample buffer. Western blotting was performed using the resuspended samples obtained from immunoprecipitation and m^7^GTP Sepharose pull-down assays.

### RNA immunoprecipitation and RNA fluorescent *in situ* hybridization (FISH)

HeLa Cells transfected with β-globin and HA-eIF4E expression constructs were harvested and lysed in hypotonic gentle lysis buffer (10 mM Tris-HCl (pH 7.5), 10 mM NaCl, 2 mM EDTA, 0.5% Triton X-100, protease inhibitor cocktail (Roche, Basel, Switzerland), 40 U/ml RNaseOUT) for 10 min on ice. Then, lysates were incubated overnight with anti-HA beads (Sigma-Aldrich). Beads were washed 10 times with washing buffer (50 mM Tris-HCl (pH 7.5), 150 mM NaCl, 0.05% Triton X-100) and resuspended in 1 ml of Trizol (Life Technologies). Protein and RNA were isolated according to the manufacturer’s protocol. For the RNA FISH experiment, HeLa cells transfected with Bglo-WT, Bglo-P39, or Bglo-P66 were fixed with 3.7% formaldehyde in PBS for 10 min and permeabilized with 70% ethanol for 1 h. Then, cells were hybridized with a β-globin mRNA-specific probe in a humidified chamber at 37 °C for 4 h. A probe targeting *GAPDH* mRNA was used as a positive control. Probe sequences targeting β-globin and *GAPDH* mRNA were 5′-(CAL Fluor Red 610)-cactcagtgtggcaaaggtg-3′ and 5′-(FAM)-gttaaaagcagccctggtga-3′, respectively. All buffers and probes used for the RNA FISH experiment were purchased from Biosearch Technologies (Novato, CA, USA). All images were obtained using a LSM700 confocal microscope (Carl Zeiss, Oberkochen, Germany).

### Statistical analysis

Data are expressed as mean ± standard deviation; P < 0.05 was considered significant. One-way ANOVA with a post-hoc test (Bonferroni) was performed to compare multiple means using SPSS for Windows (version 21.0; SPSS Inc., Armonk, NY, USA).

### Data availability statement

All data analyzed in this study are presented in this article and also available upon request.

## Results

### Some PTC-containing mRNAs expressed from constructs gBglo-P39 and gBglo-P66 are not degraded

**Figure 1:**
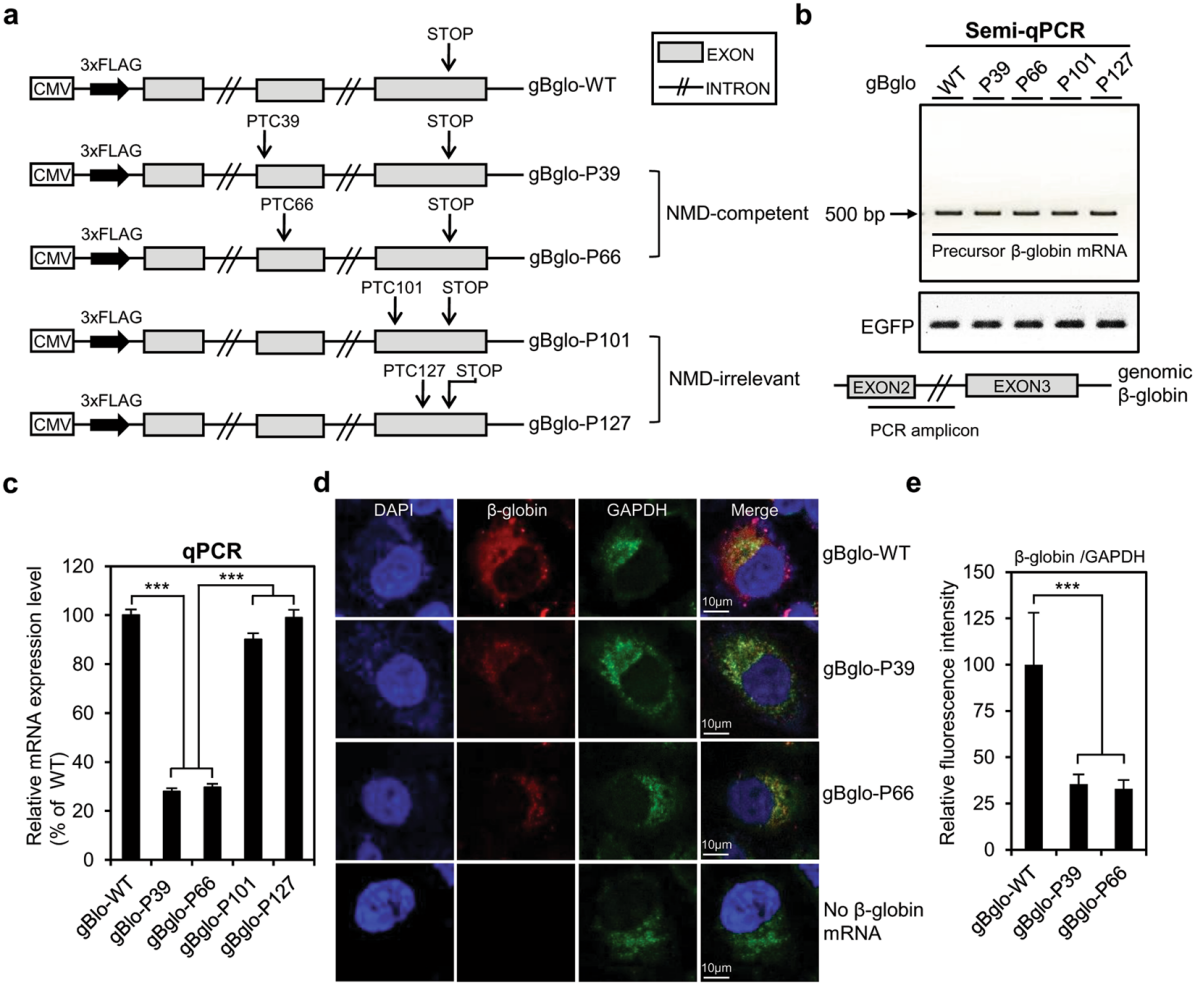
Construction and expression of vectors containing human genomic β-globin. (**a**) Schematic diagram of expression vector containing wild-type human genomic *β-globin* (gBglo-WT) and plasmids derived from gBglo-WT by introducing premature termination codons (PTCs) at the indicated positions. (**b**) Schematic of location of semi-qPCR amplicon. Semi-qPCR analysis of precursor β-globin mRNA, with EGFP serving as internal control (**c**) qPCR analysis of mRNA isolated from HeLa cells transfected with the β-globin constructs. (**d**) RNA-FISH-mediated evaluation of the cytoplasmic localization of β-globin mRNAs from gBglo-WT, gBglo-P39, and gBglo-P66. (**e**) Immunofluorescence intensity of β-globin was quantified and normalized that of *GAPDH* mRNA. Every experiment contributing to Fig. 1 was performed three independent times and the results of one representative experiment are shown. One-way ANOVA with a post-hoc test was performed to compare multiple means. ***p < 0.001. Error bars in (**c**) and (**e**) represent the SD of the mean of three independently performed qPCR analyses and the SD of the mean of normalized β-globin intensities from 10 randomly selected cells, respectively.

To study the generation of mutant proteins from PTC-containing mRNAs, we created a construct using the unspliced form of human wild-type β-globin genomic DNA (gBglo-WT). We then mutagenized gBglo-WT, introducing a PTC at amino acid position 39 or 66, to generate constructs gBglo-P39 and gBglo-P66, respectively (NMD-competent constructs). Constructs gBglo-P101 and gBglo-P127 were generated by mutagenesis of gBglo-WT, introducing a PTC at amino acid position 101 or 127, which occur in the last exon (exon 3) of the β-globin gene (NMD-irrelevant constructs) (Fig. 1a). HeLa cells were transfected with β-globin constructs and harvested 48 hours later. Semi-quantitative (q)PCR was performed to compare expression levels of precursor β-globin mRNAs and quantitative (q)PCR was performed to compare expression levels of mature β-globin mRNAs. Irrespective of PTC location, expression levels of precursor β-globin mRNAs from all β-globin constructs were similar (Fig. 1b). No significant differences were observed among the levels of mature β-globin mRNAs expressed from gBglo-WT, gBglo-P101 and gBglo-P127, which shows that the NMD-irrelevant PTC-containing gBglo-P101 and gBglo-P127 mRNAs were not recognized as NMD substrates. On the other hand, the steady-state levels of mRNA from gBglo-P39 and gBglo-P66 were approximately 30% that of gBglo-WT mRNA (Fig. 1c). RNA FISH was further performed using HeLa cells transfected with gBglo-WT, gBglo-P39, or gBGlo-P66 and immunofluorescent intensities of β-globin and *GAPDH* mRNA were quantified. The β-globin mRNAs from gBglo-WT, gBglo-P39, and gBglo-P66 showed cytoplasmic localization (Fig. 1d) and mean immunofluorescent intensities of mutant β-globin mRNA from gBglo-P39 and gBglo-P66 were approximately 35% and 33% that of gBglo-WT mRNA, respectively (Fig. 1e).

### Undegraded PTC-containing gBglo-P39 and gBglo-P66 mRNA in a steady state are as stable as their PTC-free counterparts, but generate negligible amounts of mutant protein

**Figure 2:**
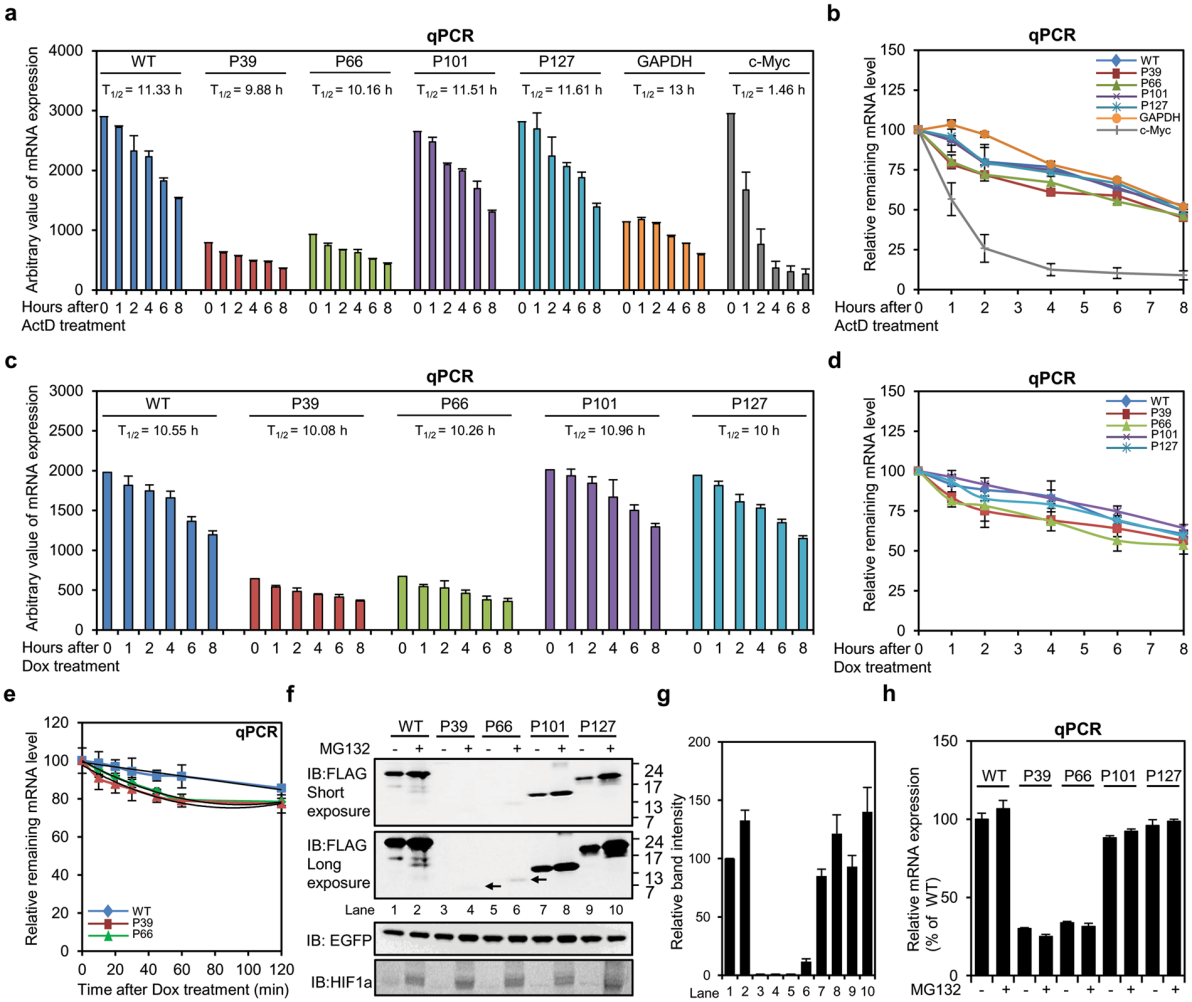
The undegraded PTC-containing mRNAs from gBglo-P39 and gBglo-P66 are highly stable, but generate trace amounts of mutant proteins in a steady state. **(a**,**b)** qPCR analysis of mRNA expressed from expression vectors gBglo-WT, gBglo-P39, gBglo-P66, gBglo-P101, and gBglo-P127 in HeLa cells at indicated times after treatment with Actinomycin D (ActD). GAPDH and c-Myc served as stable and highly unstable controls, respectively. (**c**,**d**) qPCR analysis of mRNA expressed from Tet-Off β-globin expression vectors gBglo-WT, gBglo-P39, gBglo-P66, gBglo-P101, and gBglo-P127 in HeLa cells at indicated times after treatment with Doxycycline (Dox). Arbitrary values and relative values are shown in (**a**) and (**c**), and in (**b**) and (**d**), respectively. (**e**) qPCR analysis of mRNA expressed from gBglo-WT, gBglo-P39, and gBglo-P66 at early time points after Dox treatment. (**f**) Western analysis of wild-type and mutant protein expression from the indicated genomic β-globin constructs in HeLa cells with or without proteasome inhibitor MG132. Arrows indicated the expected positions of mutant proteins. GAPDH served as internal control. HIF1α expression was measured to confirm inhibition of proteasomal degradation by MG132. (**g**) Quantification of band intensities for the western blot shown in (**f**). (**h**) qPCR analysis of mRNA expressed from the indicated genomic β-globin constructs in HeLa cells with or without MG132. Every experiment summarized in Fig. 2 was performed two independent times and the results of one representative experiment are shown. Error bars in (**a–e**) and (**h**), and in (**g**) represent the SD of the mean of two independently performed qPCR analyses and the SD of the mean of band intensities obtained from two independently performed western blots, respectively. IB, immunoblot.

Generation of mutant proteins is largely dependent on the stability of their source mRNAs. Therefore, we sought to investigate the stability of undegraded PTC-containing gBglo-P39 and gBglo-P66 mRNA in a steady state. To measure the mRNA level after the inhibition of transcription, we employed Actinomycin D, which blocks RNA polymerases. HeLa cells were transfected with the genomic β-globin constructs, treated with ActD, harvested at six time points (0, 1, 2, 4, 6, and 8 h after ActD treatment), and then analyzed by qPCR. The estimated half-lives of β-globin mRNA from gBglo-WT, gBglo-P39, gBglo-P66, gBglo-P101, and gBglo-P127 were 11.33 h, 9.88 h, 10.16 h, 11.51 h, and 11.61 h, respectively (Fig. 2a). The time-dependent decrease in mRNA expression from the five β-globin constructs was similar, regardless of PTC location (Fig. 2b). Although ActD is the most widely used reagent to estimate the RNA half-life, several studies have reported unexpected effects of ActD on RNA degradation and translation^18,19^. Therefore, we additionally generated a Tet-Off β-globin expression vector system by cloning β-globin genomic DNA into a Tet-Off vector, which allowed us to exclude any global and toxic effects of Actinomycin D on translation and RNA degradation. HeLa cells were transfected with the Tet-Off β-globin constructs, treated with Dox, harvested at the same time points used for the ActD-mediated chase experiment, and then analyzed by qPCR. The estimated half-lives of β-globin mRNA from gBglo-WT, gBglo-P39, gBglo-P66, gBglo-P101, and gBglo-P127 were 10.55 h, 10.08 h, 10.26 h, 10.96 h, and 10 h, respectively (Fig. 2c). We also found that the time-dependent decrease in the mRNA level from the five Tet-Off β-globin constructs was similar to that observed in the ActD-mediated chase experiment (Fig. 2d). Comparing the half-lives obtained using the Tet-Off system and using ActD showed that the remaining PTC-containing gBglo-P39 and gBglo-P66 mRNAs in a steady state were as stable as the wild-type β-globin mRNAs, and we considered these mRNAs a potential and stable source for the generation of mutant proteins. Transfecting gBglo-WT, gBglo-P39, and gBglo-P66 into HeLa cells, the stability of PTC-containing mRNAs from gBglo-P39 and gBglo-P66 was further measured at earlier time points after Dox treatment. Unlike wild-type β-globin mRNA, PTC-containing mRNA from gBglo-P39 and gBglo-P66 showed a biphasic decay pattern at earlier time points, which is consistent with a previous report^8^, and further demonstrated that there are two PTC-containing mRNA populations (highly unstable and stable populations) (Fig. 2e). Then, the mutant protein expression from the gBglo-P39 and gBglo-P66 constructs was analyzed in HeLa cells. Western analysis showed that the levels of mutant β-globin expressed from gBglo-P101 and gBglo-P127 were approximately 71% and 73%, respectively, that of wild-type β-globin expressed from gBglo-WT; however, mutant β-globin protein was undetectable in cells transfected with gBglo-P39 and gBglo-P66. We confirmed mRNA expression from each construct by qPCR (Supplementary Fig. S1a).

Considering the stability of the undegraded PTC-containing mRNAs from gBglo-P39 and gBglo-P66 in a steady state, the undetectable levels of protein expression from gBglo-P39 and gBglo-P66 was attributable to intrinsic low stability of the mutant proteins. To evaluate the stability of mutant β-globins, we generated cDNA forms of the original β-globin expression constructs (cBglo-WT, cBglo-P39, cBglo-P66, cBglo-P101, and cBglo-P127). These vectors expressed NMD-irrelevant PTC-containing mRNAs because of the lack of EJC assembly (Supplementary Fig. S1b). The five cDNA β-globin constructs were transfected into HeLa cells and, this time, cells were treated with proteasome inhibitor MG132 to prevent potential proteasomal degradation of unstable mutant proteins. Protein and mRNA levels were analyzed by western and qPCR, respectively. Mutant proteins expressed from cBglo-P101 and cBglo-P127 were detected with and without proteasomal inhibition. Low level of mutant protein expressed from cBglo-P39 was detected, only after MG132 treatment, and, notably, substantial level of mutant protein expressed from cBglo-P66 was detected with MG132 treatment. Expression levels of mRNAs from all cDNA β-globin constructs were almost similar (Supplementary Fig. S1c). This finding indicates that the gBglo-P66 construct is an appropriate model to investigate the generation of mutant proteins from PTC-containing mRNAs in a steady state. Then, protein and mRNA expression were analyzed in HeLa cells transfected with the genomic β-globin expression constructs. Mutant proteins expressed from gBglo-P66 were not detectable, although MG132 treatment rescued the expression of mutant protein (Fig. 2f). HIF1α expression was measured to confirm inhibition of proteasomal degradation by MG132. The intensities of β-globin bands were quantified by densitometry (Fig. 2g). We also confirmed that mRNA expression from each construct was minimally affected by MG132 treatment (Fig. 2h).

### eIF4E-dependent translation is not involved in the generation of mutant proteins from gBglo-P66

**Figure 3:**
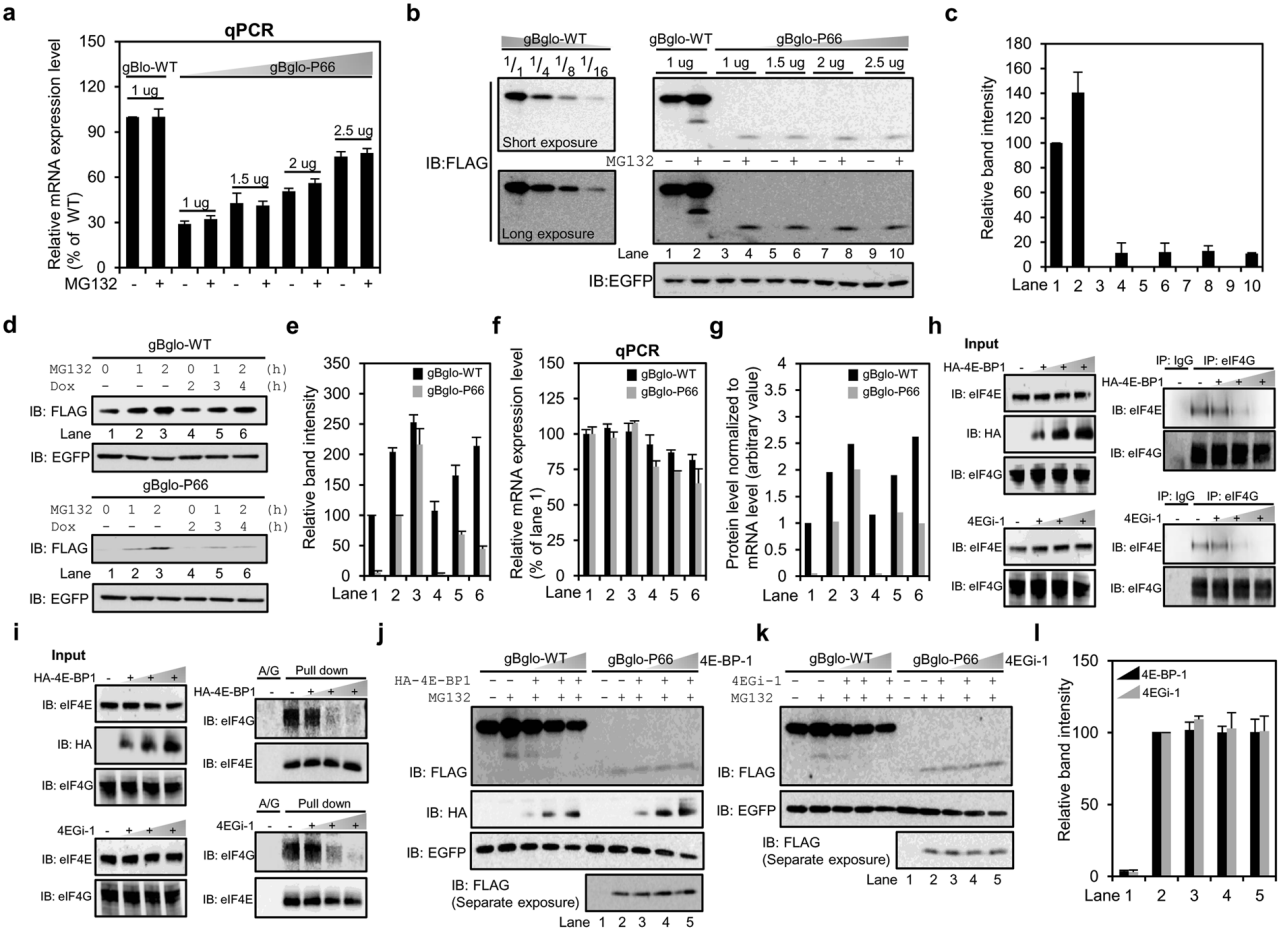
Trace amounts of mutant proteins from gBglo-P66 do not originate from eIF4E-dependent translation. (**a**) qPCR analysis of relative mRNA expression in HeLa cells transfected with constant amount of gBglo-WT or increasing amounts of gBglo-P66 in the presence and absence of MG132. (**b**) Western analysis of protein expression in the same samples used for (**a**) in the presence and absence of MG132. GAPDH served as internal control. **(c)** Quantification of band intensities for the western blot shown in (**b**). (**d**) Western analysis of protein expression in HeLa cells transfected with gBglo-WT or gBglo-P66 with and without Dox preincubation. The HeLa cells were also treated with MG132 for the indicated time. (**e**) Quantification of band intensities for the western blot shown in (**d**). (**f**) qPCR analysis of relative mRNA expression levels in the same samples used in (**d**). (**g**) Normalization of protein levels quantified in (**e**) relative to its RNA level shown in (**f**). (**h**,**i**) An immunoprecipitation assay of eIF4E/eIF4G and a pull-down assay using m^7^GTP-Sepharose after transfecting gradually increasing amounts of HA-4E-BP1 or treatment with gradually increasing concentrations of 4EGi-1. (**j**) Western analysis of protein expression in HeLa cells cotransfected with constant amount of gBglo-WT or gBglo-P66 and increasing amounts of expression vector encoding eIF4E-dependent translation inhibitors 4E-BP1. (**k**) Western analysis of protein expression in HeLa cells transfected with gBglo-WT or gBglo-P66 after treatment with increasing concentrations of 4EGi-1. (**l**) Quantification of band intensities of separately exposed western blots excised at the gBglo-P66 size shown in (**j**) and (**k**). Every experiment in Fig. 3 was performed two independent times and the results of one representative experiment are shown. Error bars in (**a**) and (**f**), and in (**c**), (**e**), and (**l**) represent the SD of the mean of two independently performed qPCR results and the SD of the mean of band intensities obtained from two independently performed western blots, respectively.

Since mutant proteins from gBglo-P66 were detectable after MG132 treatment, we hypothesized that expression of more PTC-containing mRNAs would lead to expression of more mutant proteins. Therefore, mRNA and protein levels in HeLa cells transfected with a constant amount of gBglo-WT or increasing amounts of gBglo-P66, and cultured with or without MG132, were analyzed by qPCR and western blotting, respectively. Expression of undegraded PTC-containing gBglo-P66 mRNA increased gradually as the amount of transfected gBglo-P66 increased (Fig. 3a). However, the level of mutant protein did not increase with the accumulation of mutant mRNAs (Fig. 3b,c). Concomitant increases in mRNA and protein levels were observed when we performed the same experiment using gBglo-WT (Supplementary Fig. S2). We then sought to clarify from where the small amount of mutant β-globin protein originated. It is known that the bulk of cellular protein is produced while mRNAs undergo eIF4E-dependent translation, and some cellular proteins are also generated during the pioneer round of translation (CBP-dependent)^20,21^. If the stably remaining PTC-containing mRNAs undergo steady-state rounds of translation directed by eIF4E, we expected continuous generation of mutant proteins without influx of newly synthesized PTC-containing mRNAs. To test our hypothesis, Tet-Off β-globin expression vectors were transfected into HeLa cells to measure the changes in RNA and protein levels in gBglo-WT and gBglo-P66 over time, with and without MG132 after 2 hours of Dox preincubation, which is sufficient for the removal of most rapidly degraded PTC-containing mRNA according to our data shown in Fig. 2a–e (Fig. 3d–f). Then, the quantified protein level was normalized against its RNA level (Fig. 3g). The western blot and qPCR analyses showed that the gBglo-WT level gradually increased, with or without transcriptional inhibition in the presence of MG132, while no gradual increase in the gBglo-P66 level was observed with transcriptional inhibition in the presence of MG132. These findings indicate that the continuous influx of PTC-containing mRNAs, at least in part, contributes to the constant generation of trace amounts of mutant protein from gBglo-P66. Then, we sought to elucidate the involvement of eIF4E-dependent translation in the generation of mutant proteins from gBglo-P66 to determine which mode of translation (CBP-dependent and eIF4E-dependent mode of translation) is important for the generation of mutant proteins from gBglo-P66. We used 4E-BP1 and 4EGi-1 as specific inhibitors of eIF4E-dependent translation^17^. A coimmunoprecipitation assay of eIF4G/eIF4E and a pull-down assay using m^7^GTP-Sepharose were performed to evaluate whether 4E-BP1 and 4EGi-1 effectively inhibit eIF4E from forming an initiation complex for eIF4E-dependent translation in our system. These assays showed that the interaction of eIF4E with eIF4G was substantially disturbed when cells were transfected with gradually increasing amounts of the HA-4E-BP1 expression vector or treated with gradually increasing concentrations of 4EGi-1 (Fig. 3h,i). Then, HeLa cells were cotransfected with 4E-BP1 construct and gBglo-WT or gBglo-P66. Wild-type β-globin expression from gBglo-WT was gradually reduced as 4E-BP1 expression increased; however, mutant β-globin expression from gBglo-P66 was not affected by 4E-BP1 expression level (Fig. 3j). In parallel experiments in which transfected cells were treated with eIF4E-specific translation inhibitor 4EGi-1, we confirmed that mutant β-globin expression from gBglo-P66 was not affected by inhibition of eIF4E-dependent translation (Fig. 3k). Due to the very low expression level of gBglo-P66 compared to that of gBglo-WT in the same western blot, separately exposed blots excised at the size of gBglo-P66 (shown in Fig. 3j,k) were quantified, and these results further confirmed that the inhibition of eIF4E-dependent translation had minimal effect on mutant β-globin expression from gBglo-P66 (Fig. 3l). These findings suggest that mutant β-globin expression from gBglo-P66 is independent of eIF4E-dependent translation and could be attributed to the pioneer round of translation of newly synthesized PTC-containing mRNAs.

### Polysome association of PTC-containing mRNA expressed from gBglo-P66 is induced by inhibition of NMD

**Figure 4:**
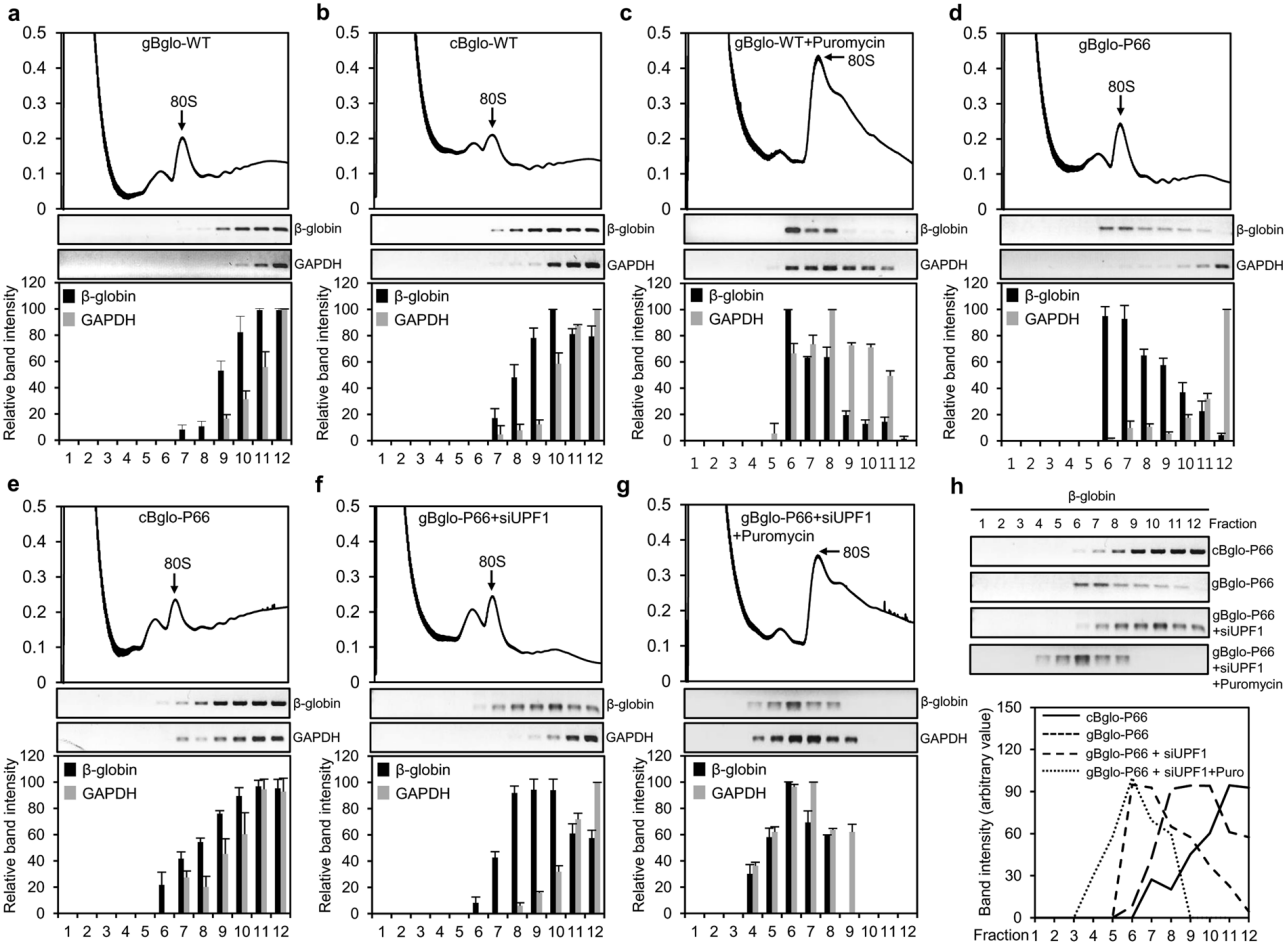
Polysome associated PTC-containing mRNAs expressed from gBglo-P66 are induced by UPF1 depletion. (**a**,**g**) Absorbance at 254 nm (upper panels) and semi-qPCR analysis of relative mRNA levels (lower panels) of sucrose gradient fractions containing polysomes of HeLa cells transfected with the indicated constructs in the absence (**a**–**e**) or presence (f and g) of siRNA targeting UPF1. Cells were treated with puromycin before they were harvested (**c**–**g**). (**h**) Comparison of distribution patterns of PTC-containing mRNAs in cells transfected with the indicated constructs in the presence and absence of UPF1 knockdown and shown in panels d–g. mRNA bands obtained from semi-qPCR were quantified by densitometry. Every experiment in Fig. 4 was performed two independent times and the results of one representative experiment are shown. Error bars represent the SD of the mean of semi-qPCR results using samples obtained from the two independently performed polysome analyses.

To evaluate the translational status of the undegraded PTC-containing mRNAs from gBglo-P66 in a steady state, HeLa cells transfected with constructs gBglo-P66 and gBglo-WT were lysed and fractionated by sucrose gradient density centrifugation. Absorbance of fractions at 254 nm was measured to identify fractions containing polysomes, which were then analyzed by Semi-qPCR. Samples prepared from cells transfected with cBglo-P66 and cBglo-WT served as NMD-irrelevant controls. We also analyzed the association of PTC-containing mRNAs from gBglo-P66 with polysomes when NMD was inhibited by siRNA-mediated UPF1 depletion. The polysome analysis showed that the gBglo-WT and cBglo-WT mRNAs were detected primarily in polysome-associated fractions (right shifted), as was GAPDH mRNA, a translationally competent control mRNA (Fig. 4a,b). The gBglo-WT mRNAs detected in polysome-associated fractions were shifted to the monosome fractions by puromycin treatment, which releases ribosomes during the elongation step, ensuring that the mRNAs detected in the heavy fractions represent polysome-associated mRNAs (Fig. 4c). The distribution pattern of β-globin gBglo-P66 mRNA was shifted left relative to that of cBglo-P66 mRNA, indicating that the undegraded PTC-containing mRNAs expressed from gBglo-P66 in a steady state were mostly associated with monosomes (left shifted) (Fig. 4d,e). On the other hand, polysome-association of gBglo-P66 mRNAs (right-shifted) was observed when NMD was inhibited by UPF1 depletion and their association with polysomes was disrupted by puromycin treatment (Fig. 4f,g). Comparing the polysome association patterns of β-globin mRNAs from gBglo-P66, cBglo-P66, and gBglo-P66 with UPF1 depletion and gBglo-P66 with both UPF1 depletion and puromycin treatment suggests that the undegraded PTC-containing mRNAs from gBglo-P66 are not efficiently translated in a steady state and that inhibition of NMD induces the association of PTC-containing mRNAs from gBglo-P66 with polysomes, which, in turn, might lead to bulk generation of mutant protein from gBglo-P66 (Fig. 4h).

### The bulk of mutant protein is generated from gBglo-P66 when NMD is inhibited

**Figure 5:**
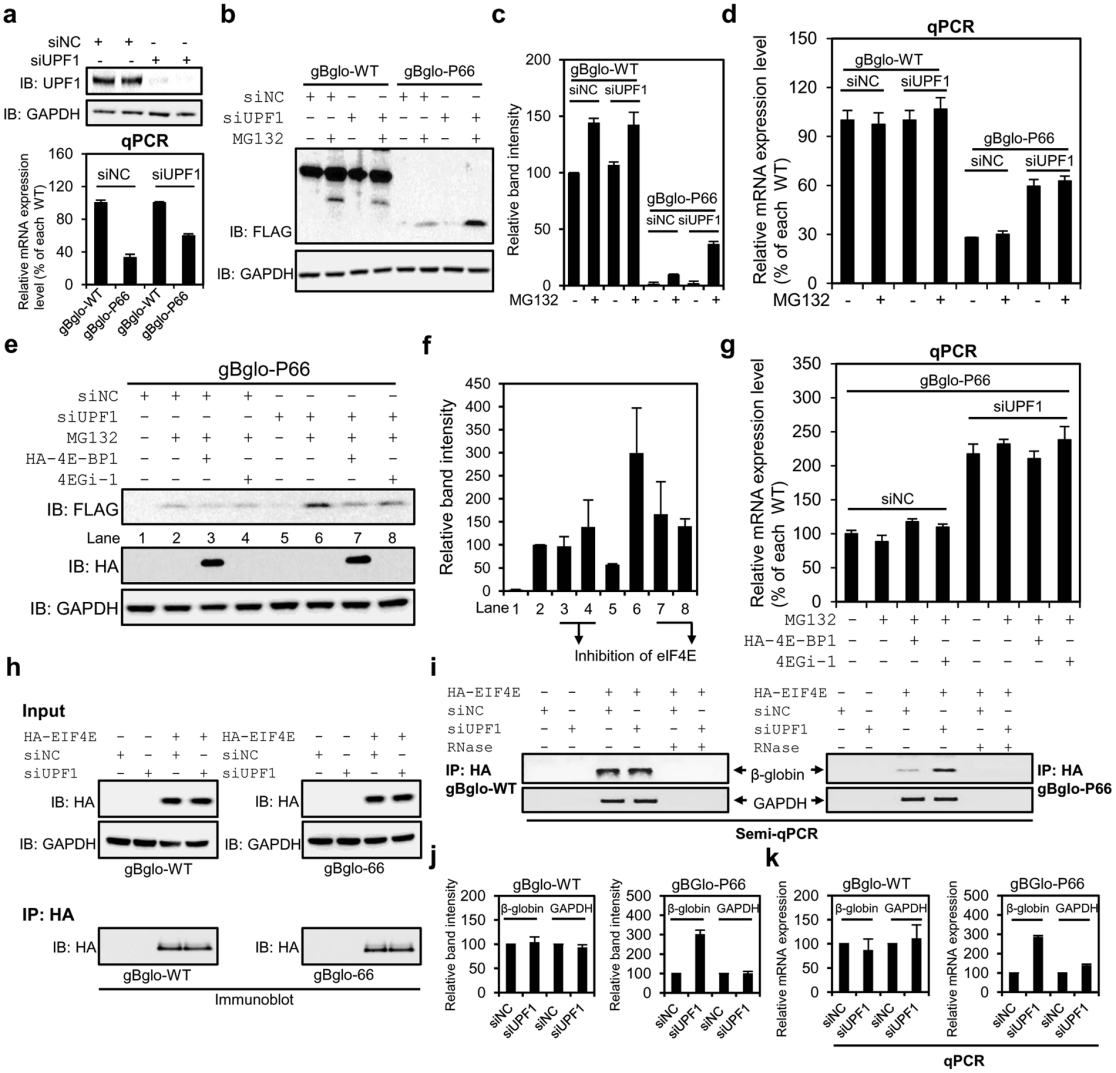
The bulk of mutant gBglo-P66 protein is generated via eIF4E-dependent translation when NMD is inhibited by UPF1 depletion. (**a**) Western analysis of UPF1 expression (upper) and qPCR analysis of β-globin mRNA level (lower) in HeLa cells transfected with siRNA targeting UPF1 or control siRNA (NC) and gBglo-WT or gBglo-P66 constructs. (**b**) Western analysis of wild-type and mutant β-globin expression in cells transfected with the indicated constructs in the presence or absence of siRNA targeting UPF1, in the presence or absence of MG132. (**c**) Quantification of band intensities for the western blot shown in (**b**). (**d**) qPCR analysis of mRNA levels in the same samples used in (**b**). (**e**) Western analysis of mutant β-globin translated from NMD-rescued PTC-containing gBglo-P66 mRNAs in HeLa cells transfected with gBglo-P66 with or without cotransfection of construct encoding HA-4E-BP1, in the presence or absence of siRNA targeting UPF1 (siUPF1) or control siRNA (siNC), and in the presence and absence of MG132 and 4EGi-1. (**f**) Quantification of band intensities for the western blot shown in (**e**). (**g**) qPCR analysis of mRNA expression levels in cells analyzed in (**e**). (**h**) RNA immunoprecipitation assays were performed using HeLa cells transfected with a HA-eIF4E vector with gBglo-WT or gBglo-P66 when UPF1 was depleted or not. RNA immunoprecipitation using cells transfected with HA-containing empty vector was performed as a negative control experiment. (**i**) eIF4E-bound β-globin mRNA level was analyzed by semi-qPCR in presence and in absence of RNase. (**j**) Quantification of band intensities for the semi-qPCR analysis shown in (**i**). (**k**) qPCR analysis of eIF4E-bound β-globin mRNA levels. Every experiment in Fig. 5 was performed two independent times and the results of one representative experiment are shown. Error bars in (**a**), (**d**), (**g**), and (**k**), in (**c**), (**f**), and in (**j**) represent the SD of the mean of two independently performed qPCR results and the SD of the mean of band intensities obtained from two independently performed western blots, and the SD of the mean of band intensities obtained from two independently performed semi-qPCR results, respectively. Control siRNA (siNC).

Based on the results of polysome analysis, we further determined if the bulk of mutant protein was generated from gBglo-P66 when NMD was inhibited. Therefore, levels of protein and mRNA expression from gBglo-WT and gBglo-P66 were measured with and without NMD inhibition. To rule out repressive effects on translation, chemical NMD inhibitors, such as cycloheximide and emetine, were not used in this study. qPCR analysis showed that gBglo-P66 mRNA expression level nearly doubled when NMD was inhibited by UPF1 depletion, whereas gBglo-WT mRNA expression was essentially unchanged (Fig. 5a). Concomitant western analysis of protein levels showed that only slight increase in gBglo-WT protein was associated with UPF1 depletion (Fig. 5b and c). In addition, gBglo-P101 and gBglo-P127 (NMD-irrelevant) mRNA and protein levels were also barely affected by NMD inhibition (Supplementary Fig. S3). On the other hand, UPF1 knockdown induced about 4-fold increase in mutant gBglo-P66 protein level in the presence of MG132 when gBglo-P66 mRNA expression level doubled (Fig. 5b–d). To test the involvement of eIF4E-dependent translation in the increase in mutant proteins by UPF1 depletion, we measured the mutant gBglo-P66 protein level after inhibition of eIF4E-dependent translation by 4E-BP1 or 4EGi-1 with and without UPF1 depletion. Western analysis showed that increased level of mutant gBglo-P66 protein seen with UPF1 downregulation in the presence of MG132 was repressed by either 4E-BP1 overexpression or 4EGi-1 treatment (Fig. 5e,f). The level of gBglo-P66 mRNA was not affected by cotransfection of 4E-BP1 or 4EGi-1 treatment (Fig. 5g). RNA immunoprecipitation assays were further performed using HeLa cells transfected with gBglo-WT or gBglo-P66 when UPF1 was depleted or not. Immunoprecipitation using cells transfected the HA-containing empty vector was performed as a negative control (Fig. 5h). After immunoprecipitation by HA, eIF4E-bound mRNAs from gBglo-WT and gBglo-P66 were extracted and used for subsequent semi-qPCR. Samples treated with RNase were used as a negative control for semi-qPCR (Fig. 5i). Semi-qPCR results were quantified by densitometry (Fig. 5j). qPCR was further performed using the same samples used for semi-qPCR to precisely measure the increase in eIF4E-bound β-globin mRNAs by UPF1 depletion (Fig. 5k). Both semi-qPCR and qPCR analyses showed that eIF4E-bound mutant β-globin mRNA from gBglo-P66 increased (~3-fold) after UPF1 depletion, while eIF4E-bound wild-type β-globin mRNA from gBglo-WT changed very little. These findings provide evidence that eIF4E-directed translation is involved in generating the bulk of mutant proteins from gBglo-P66 when NMD is inhibited.

### Depletion of UPF1 or SMG1 is necessary for the generation of the bulk of mutant proteins

**Figure 6:**
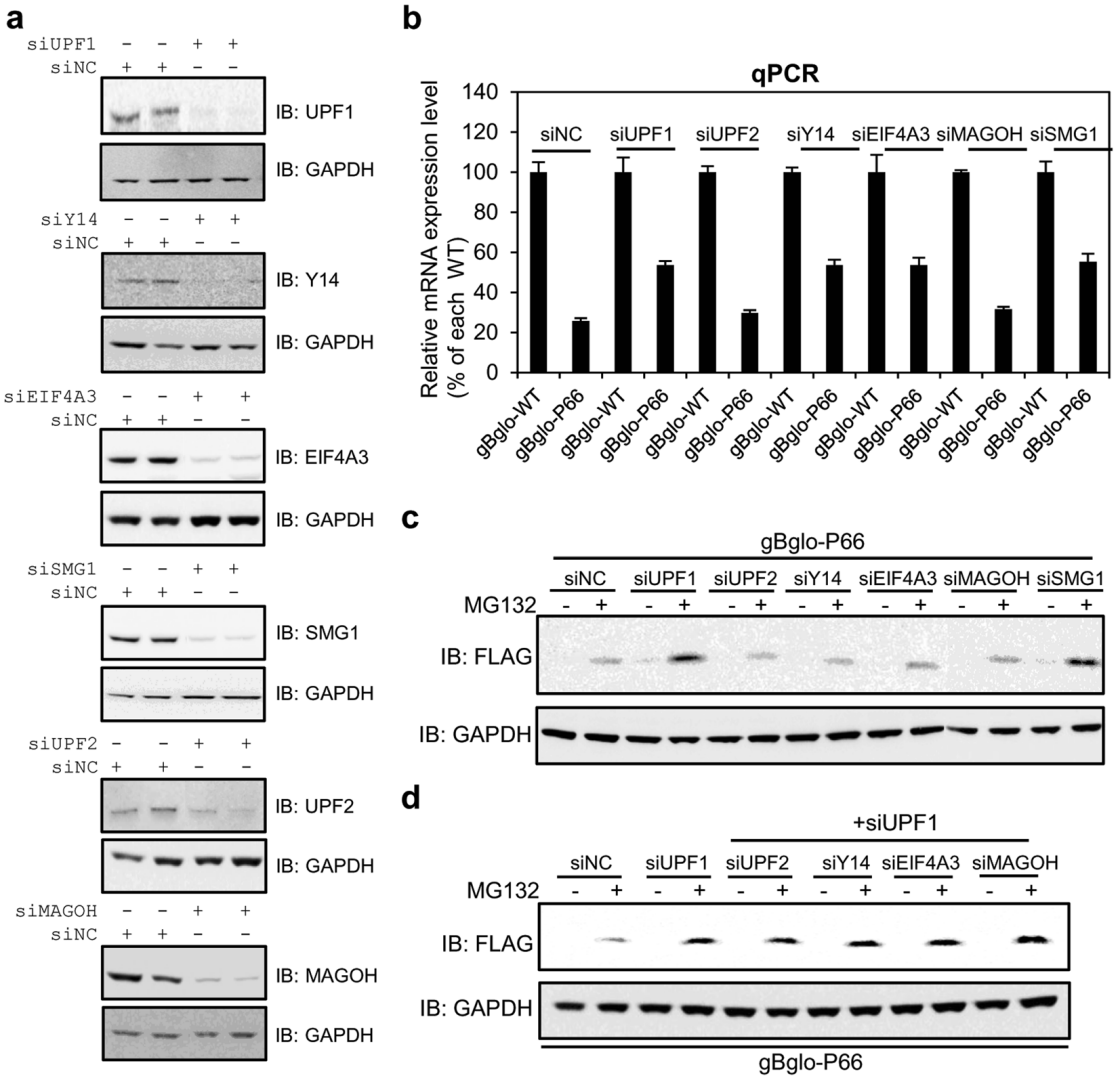
Inhibition of UPF1 or SMG-1 is required for generation of bulk of mutant protein from NMD-rescued PTC-containing gBglo-P66 mRNA. (**a**) Western analysis of siRNA mediated downregulation of core NMD and EJC factors UPF1, Y14, EIF4A3, SMG1, UPF2, and MAGOH in HeLa cells. GAPDH served as internal control. (**b**) qPCR analysis of relative β-globin mRNA levels in HeLa cells transfected with gBglo-WT or gBglo-P66 and siRNA against each factor. (**c**) Western analysis of β-globin derived from NMD-rescued PTC-containing gBglo-P66 mRNA in the same samples used in (**b**). (**d**) Concomitant inhibition of UPF1 with UPF2, Y14, EIF4A3, or MAGOH in HeLa cells transfected with gBglo-P66 in the presence and absence of MG132. Every experiment in Fig. 6 was performed two independent times and the results of one representative experiment are shown. Error bars in (**a**) represent the SD of the mean of two independently performed qPCR results.

We used siRNA targeting UPF1 to inhibit NMD and found that NMD inhibition significantly increased the level of mutant protein. However, it was not clear whether inhibition of NMD by depletion of any factors involved in NMD is sufficient to generate the bulk of mutant protein. To determine if depletion of NMD factors or EJC components would lead to the generation of bulk mutant from gBglo-P66, core NMD factors (UPF1, UPF2 and SMG1) and core EJC components (Y14, EIF4A3 and MAGOH) were downregulated (Fig. 6a), and levels of gBglo-P66 mRNA and protein were assessed in HeLa cells transfected with gBglo-P66 and siRNA against each factor. The increase (~2-fold) in gBglo-P66 mRNA level was similarly observed when UPF1, Y14, siEIF4A3, and SMG1 were downregulated. A slight increase in gBglo-P66 mRNA was observed after downregulation of UPF2 and MAGOH (Fig. 6b). The level of mutant protein from gBglo-P66 was significantly increased upon downregulation of UPF1 or SMG1, but not the other factors (Fig. 6c). Moreover, concomitant downregulation of UPF1 with UPF2, Y14, EIF4A3, or MAGOH did not lead to synergistic increases in mutant protein levels, compared to single knockdown of UPF1 (Fig. 6d). These findings suggest that downregulation of NMD or EJC factors commonly inhibits NMD and that inhibition of UPF1 or SMG1 is further required for the generation of the bulk of mutant proteins.

## Discussion

NMD is a well-studied post-transcriptional mechanism that degrades PTC-containing mRNAs to prevent generation of mutant proteins^22^. Although NMD efficiently removes PTC-containing mRNAs, recent studies have provided evidence that some PTC-containing mRNAs escape NMD, whereas others are immuned to NMD^8,23^. If these mutant mRNAs are translated, truncated mutant proteins with neopeptides are generated^7^.

The generation of mutant proteins is directly linked to development of diseases, including cancers, and mutant proteins have also been utilized in developing treatments for diseases. Therefore, it is becoming more important to understand whether PTC-containing mRNAs could be used as a source for generation of mutant protein and specify factors regulating the generation of mutant protein. We and other groups previously reported that most NMD-irrelevant PTC-containing mRNA is a potent source of truncated mutant proteins containing neopeptides^7,24^. However, mRNAs with NMD-irrelevant PTCs represent only a small proportion of all PTC-containing mRNAs. Further, NMD is dynamically regulated by various external or internal conditions such as hypoxia, serum starvation, heat shock, and regulation of NMD factors, suggesting there is a strong possibility that PTC-containing mRNAs are not degraded and evade NMD^9^.

We herein addressed whether mutant proteins are generated from PTC-containing mRNAs depending on NMD status. Using general and Tet-Off β-globin expression constructs, we demonstrated that the undegraded PTC-containing mRNAs (previously reported as a NMD-resistant population^8^) expressed from gBglo-P39 and gBglo-P66 were as stable as wild-type gBglo-WT mRNAs in a steady state, and these NMD-resistant PTC-containing gBglo-P66 mRNA gradually accumulated as the amount of gBglo-P66 plasmid transfected increased. These findings show the possibility that NMD has only a limited capacity to remove PTC-containing mRNAs diffusing away from a region of degradation that is proximal to the nuclear envelope^8^. Despite their stable expression, NMD-resistant PTC-containing mRNAs expressed from gBglo-P66 were enriched in monosomes, and their accumulation barely contributed to generation of mutant protein. With continuous influx of PTC-containing mRNAs, a trace amount of mutant gBglo-P66 protein was detected after proteasome inhibition, and eIF4E-dependent translation was not involved in this process. These findings may suggest 1) that NMD-resistant PTC-containing mRNAs from gBglo-P66 no longer undergo multiple rounds of translation before their degradation and 2) that mutant proteins are generated during the pioneer round of translation of newly synthesized PTC-containing mRNAs. However, these mRNAs should be further characterized to understand a turn-over mechanism of NMD-resistant PTC-containing mRNAs after their release from the nucleus.

After the pioneer round of translation, the 5′-m7GpppN cap structure binds to eIF4E. Based on the fact that eIF4E-bound mRNAs lack association with EJC components, eIF4E-bound PTC-containing mRNAs are considered immune to NMD^25^. eIF4E directs steady-state rounds of mRNA translation, and the translation of eIF4E-bound mRNAs generates the bulk of cellular proteins^9^. Our data demonstrate that inhibition of NMD by UPF1 depletion leads to a significant increase of mutant proteins from gBglo-P66, and eIF4E-dependent translation is involved in this process. Supporting these data, UPF1 depletion induced polysome association of PTC-containing mRNAs from gBglo-P66. These findings allow us to consider two possible models: One is that monosome-enriched NMD-resistant PTC-containing mRNAs in a steady state might start to associate with polysomes by UPF1 depletion. The other is that the PTC-containing mRNAs rescued from NMD by UPF1 knockdown bind to eIF4E and undergo multiple rounds of translation. Considering that both monosome and polysome-enriched PTC-containing mRNAs were observed when UPF1 was depleted, we believe that the latter model is more plausible. Rigorous work will be needed to understand differences between NMD-resistant PTC-containing mRNAs and the NMD-rescued PTC-containing mRNAs.

With differential inhibitory effect on NMD, downregulation of NMD and EJC factors induced an increase of PTC-containing mRNAs from gBglo-P66. Importantly, inhibition of NMD by downregulation of UPF1 or SMG1 selectively resulted in increases in both PTC-containing mRNAs and truncated mutant proteins. Generally, UPF1 is known to interact with eRF1, eRF3, and SMG1 to form the SURF complex, which subsequently binds to the EJC complex and mRNA decay factors, including SMG5, SMG6, and SMG7, for mRNA degradation^26^. It has been also reported that UPF1 phosphorylated by SMG1 interacts with eIF3, which further suppresses translation of PTC-containing mRNAs by inhibiting the eIF3-dependent conversion of 40 S/Met-tRNA_i_^Met^/mRNA to translationally competent 80 S/Met-tRNA_i_^Met^/mRNA initiation complexes to repress continued translation initiation^27^. These previous reports and our data imply that UPF1 plays suppressive roles in translation of PTC-containing mRNAs even after NMD inhibition mediated by EJC knockdown, and depletion of UPF1 is required for bulk generation of mutant proteins. Since both formation of SURF complex and phosphorylation of UPF1 after the association with EJC require SMG1, we assume that SMG1 plays supplementary roles in UPF1-mediated translational repression.

In the past decades, many studies have extensively searched for mutations in various cancers, such as colon, breast, brain, and pancreatic tumors^28–30^. Among the mutations in cancer, some are truncating mutations (nonsense or frameshift mutations) that lead to generation of PTC-containing mRNAs. Our findings provide evidence that trace amounts of mutant proteins are constantly generated in the pioneer round of translation of PTC-containing mRNAs, and a large amount of mutant proteins can be generated by inhibition of NMD. Then, the truncated mutant proteins are eventually degraded by the proteasome system (Supplementary Fig. S4). These findings support the rationale of recent immunotherapeutic approaches based on the generation and proteasomal degradation of mutant proteins with neoantigens^31–34^. Moreover, Pastor *et al*. experimentally showed that tumors expressing mutant proteins from a β-globin construct that also encoded ovalbumin peptides were effectively eliminated by CD8+ T cells when NMD was inhibited^35^. Stimulating the immune system for treatment of tumors is not a new idea. Many studies have observed intensive immune infiltration around tumor tissues, and now it is important to identify recurrent neoantigens that can be used as specific targets by activated T cells^36,37^. We, therefore, suggest that PTC-containing mRNAs are continuous and manipulable sources of mutant proteins, and appropriate application of the mutant proteins will be beneficial in diagnosis or treatment of various cancers.

## Electronic supplementary material

Supplementary Data

## References

[1] Brogna S, Wen J (2009). Nonsense-mediated mRNA decay (NMD) mechanisms. Nat Struct Mol Biol.

[2] Le Hir H, Moore MJ, Maquat LE (2000). Pre-mRNA splicing alters mRNP composition: evidence for stable association of proteins at exon-exon junctions. Genes Dev.

[3] Maquat LE (1995). When cells stop making sense: effects of nonsense codons on RNA metabolism in vertebrate cells. RNA.

[4] Le Hir H, Izaurralde E, Maquat LE, Moore MJ (2000). The spliceosome deposits multiple proteins 20-24 nucleotides upstream of mRNA exon-exon junctions. EMBO J.

[5] Metze S, Herzog VA, Ruepp MD, Mühlemann O (2013). Comparison of EJC-enhanced and EJC-independent NMD in human cells reveals two partially redundant degradation pathways. RNA.

[6] Holbrook JA, Neu-Yilik G, Hentze MW, Kulozik AE (2004). Nonsense-mediated decay approaches the clinic. Nat Genet.

[7] Kim WK (2013). Identification and selective degradation of neopeptide-containing truncated mutant proteins in the tumors with high microsatellite instability. Clin Cancer Res.

[8] Trcek T, Sato H, Singer RH, Maquat LE (2013). Temporal and spatial characterization of nonsense-mediated mRNA decay. Genes Dev.

[9] Maquat LE, Tarn WY, Isken O (2010). The pioneer round of translation: features and functions. Cell.

[10] Cheng J, Maquat LE (1993). Nonsense codons can reduce the abundance of nuclear mRNA without affecting the abundance of pre-mRNA or the half-life of cytoplasmic mRNA. Mol Cell Biol.

[11] Belgrader P, Cheng J, Maquat LE (1993). Evidence to implicate translation by ribosomes in the mechanism by which nonsense codons reduce the nuclear level of human triosephosphate isomerase mRNA. Proc Natl Acad Sci USA.

[12] Lejeune F, Li X, Maquat LE (2003). Nonsense-mediated mRNA decay in mammalian cells involves decapping, deadenylating, and exonucleolytic activities. Mol Cell.

[13] Barbier J (2007). Regulation of H-ras splice variant expression by cross talk between the p53 and nonsense-mediated mRNA decay pathways. Mol Cell Biol.

[14] Rio Frio T (2008). Premature termination codons in PRPF31 cause retinitis pigmentosa via haploinsufficiency due to nonsense-mediated mRNA decay. J Clin Invest.

[15] Van Allen EM (2015). Genomic correlates of response to CTLA-4 blockade in metastatic melanoma. Science.

[16] Le DT (2015). PD-1 Blockade in Tumors with Mismatch-Repair Deficiency. N Engl J Med.

[17] Moerke NJ (2007). Small-molecule inhibition of the interaction between the translation initiation factors eIF4E and eIF4G. Cell.

[18] Schwanhäusser B (2011). Global quantification of mammalian gene expression control. Nature.

[19] Bensaude O (2011). Inhibiting eukaryotic transcription: Which compound to choose? How to evaluate its activity?. Transcription.

[20] Apcher S, Manoury B, Fåhraeus R (2012). The role of mRNA translation in direct MHC class I antigen presentation. Curr Opin Immunol.

[21] Apcher S (2011). Major source of antigenic peptides for the MHC class I pathway is produced during the pioneer round of mRNA translation. Proc Natl Acad Sci USA.

[22] Shoemaker CJ, Green R (2012). Translation drives mRNA quality control. Nat Struct Mol Biol.

[23] You KT (2007). Selective translational repression of truncated proteins from frameshift mutation-derived mRNAs in tumors. PLoS Biol.

[24] Kang JQ, Shen W, Macdonald RL (2009). Two molecular pathways (NMD and ERAD) contribute to a genetic epilepsy associated with the GABA(A) receptor GABRA1 PTC mutation, 975delC, S326fs328X. J Neurosci.

[25] Isken O, Maquat LE (2008). The multiple lives of NMD factors: balancing roles in gene and genome regulation. Nat Rev Genet.

[26] Garneau NL, Wilusz J, Wilusz CJ (2007). The highways and byways of mRNA decay. Nat Rev Mol Cell Biol.

[27] Isken O (2008). Upf1 phosphorylation triggers translational repression during nonsense-mediated mRNA decay. Cell.

[28] Vogelstein B (2013). Cancer genome landscapes. Science.

[29] Weinstein JN (2013). The Cancer Genome Atlas Pan-Cancer analysis project. Nat Genet.

[30] Ciriello G (2013). Emerging landscape of oncogenic signatures across human cancers. Nat Genet.

[31] Desrichard, A., Snyder, A. & Chan, T. A. Cancer Neoantigens and Applications for Immunotherapy. *Clin Cancer Res*, 10.1158/1078-0432.CCR-14-3175 (2015). 10.1158/1078-0432.CCR-14-3175 26515495

[32] Gubin MM, Artyomov MN, Mardis ER, Schreiber RD (2015). Tumor neoantigens: building a framework for personalized cancer immunotherapy. J Clin Invest.

[33] Maby P (2015). Correlation between Density of CD8+ T-cell Infiltrate in Microsatellite Unstable Colorectal Cancers and Frameshift Mutations: A Rationale for Personalized Immunotherapy. Cancer Res.

[34] Schumacher TN, Schreiber RD (2015). Neoantigens in cancer immunotherapy. Science.

[35] Pastor F, Kolonias D, Giangrande PH, Gilboa E (2010). Induction of tumour immunity by targeted inhibition of nonsense-mediated mRNA decay. Nature.

[36] Dolcetti R (1999). High prevalence of activated intraepithelial cytotoxic T lymphocytes and increased neoplastic cell apoptosis in colorectal carcinomas with microsatellite instability. Am J Pathol.

[37] Alexander J (2001). Histopathological identification of colon cancer with microsatellite instability. Am J Pathol.

