# Supplementary material for: mRNAs containing NMD-competent premature termination codons are stabilized and translated under UPF1 depletion
Source: Sci Rep. 2017 Nov 20;7:15833. doi: 10.1038/s41598-017-16177-9 (PMC5696521; doi:10.1038/s41598-017-16177-9)
Supplement: Supplementary file 1 — Supplementary Data [file 41598_2017_16177_MOESM1_ESM.pdf]

mRNAs containing NMD-competent premature termination codons are stabilized and translated under UFP1 depletion

Won Kyu Kim[1], SeongJu Yun[1], Yujin Kwon[1], Kwon Tae You[2], Nara Shin[3], Jiyoon Kim[4], and Hoguen Kim[1]*

1. Department of Pathology and Brain Korea 21 PLUS Projects for Medical Science, Yonsei University College of Medicine, Seoul 03722, Republic of Korea

2. Broad Institute of MIT and Harvard, Cambridge, MA 02142, USA

3. Department of Genetics, Harvard Medical School, 77 Avenue Louis Pasteur, Boston, MA 02115, USA

4. Department of Pharmacology and Brain Korea 21 Project for Medical Sciences, Yonsei University College of Medicine, Seoul 03722, Republic of Korea

*Correspondence:

Hoguen Kim, M.D., Ph.D.

Professor of Pathology

Yonsei University College of Medicine

Seoul 120-752, Korea

Tel: +82 2 2228 1761/ Fax: +82 2 363 5263/ e-mail: hkyonsei@yuhs.ac

Supplementary Information

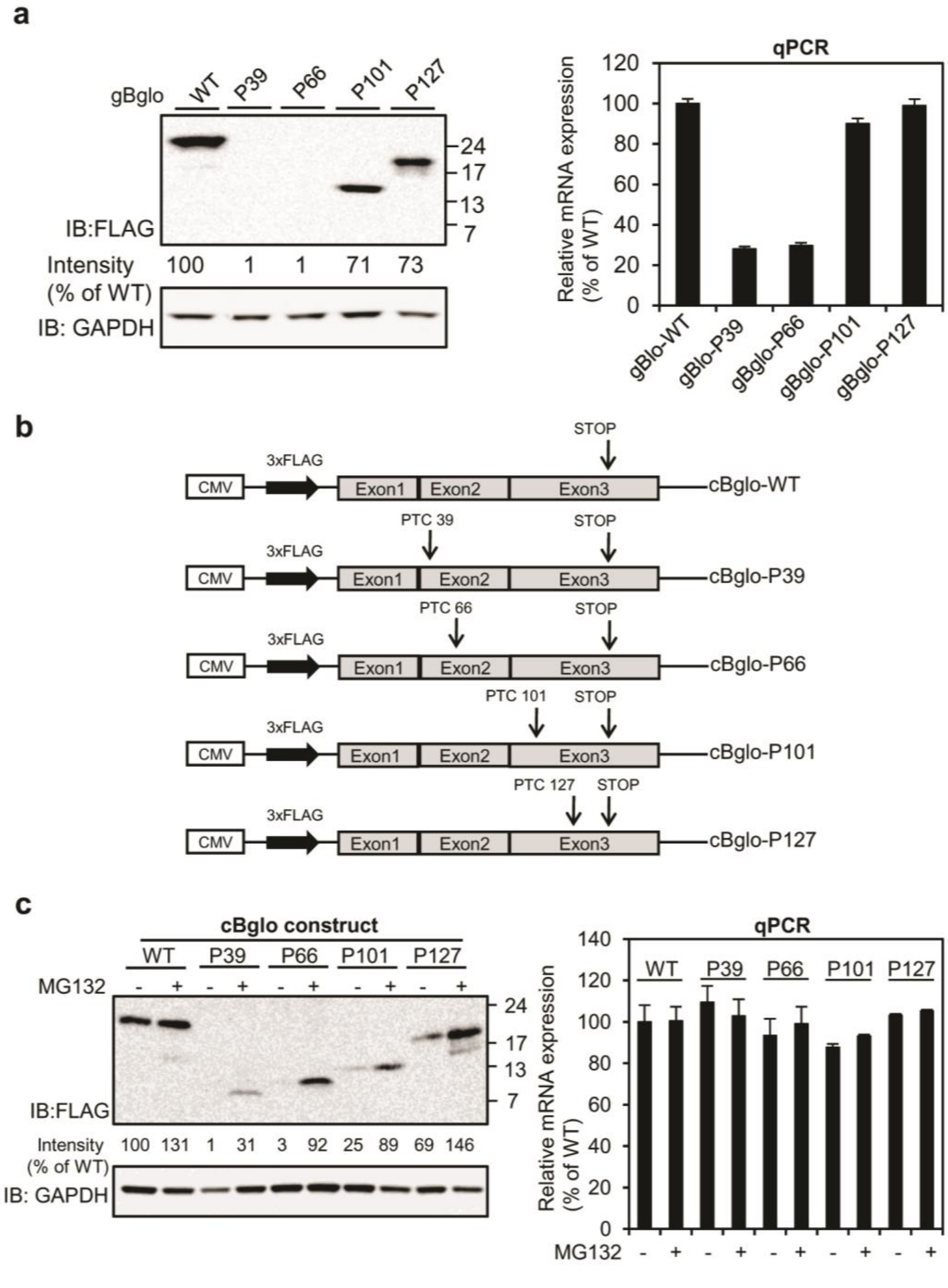

Supplementary Figure S1. The genomic human β-globin expression construct gBglo-P66 is a suitable model to evaluate generation of mutant proteins from NMD-resistant PTC-containing mRNAs. **(a)** Western analysis of protein expression using anti-FLAG antibody (left) and qPCR analysis of mRNA expression (right) in HeLa cells transfected with the indicated genomic β-globin constructs. IB, immunoblot. Band density relative to wild-type is expressed numerically below each lane of blot. GAPDH served as internal control. **(b)** Schematic diagram of expression vector containing wild-type human β-globin cDNA (cBglo-WT) and plasmids derived from cBglo-WT by mutagenesis to introduce premature termination codons (PTCs) at the indicated positions. **(c)** (Left) Western analysis of wild-

type and mutant protein expression from the indicated β-globin cDNA constructs in HeLa cells in the presence and absence of proteasome inhibitor MG132. Arrow indicates the expected position of mutant protein. IB, immunoblot. Band density relative to wild-type is expressed numerically below each lane. GAPDH served as internal control. (Right) qPCR analysis of mRNA expressed from the indicated β-globin cDNA constructs in the presence and absence of MG132.

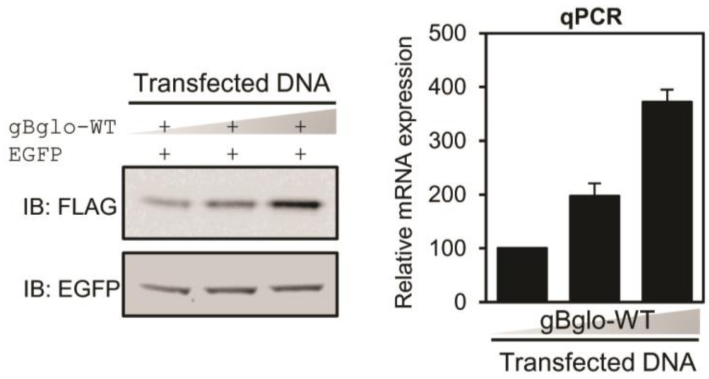

Supplementary Figure S2. Overexpression of gBglo-WT induces accumulation of both β-globin mRNA and protein. HeLa cells were transfected with increasing amounts of gBglo-WT. Western analysis of protein expression (left) and qPCR analysis of mRNA expression level (right) in HeLa cells.

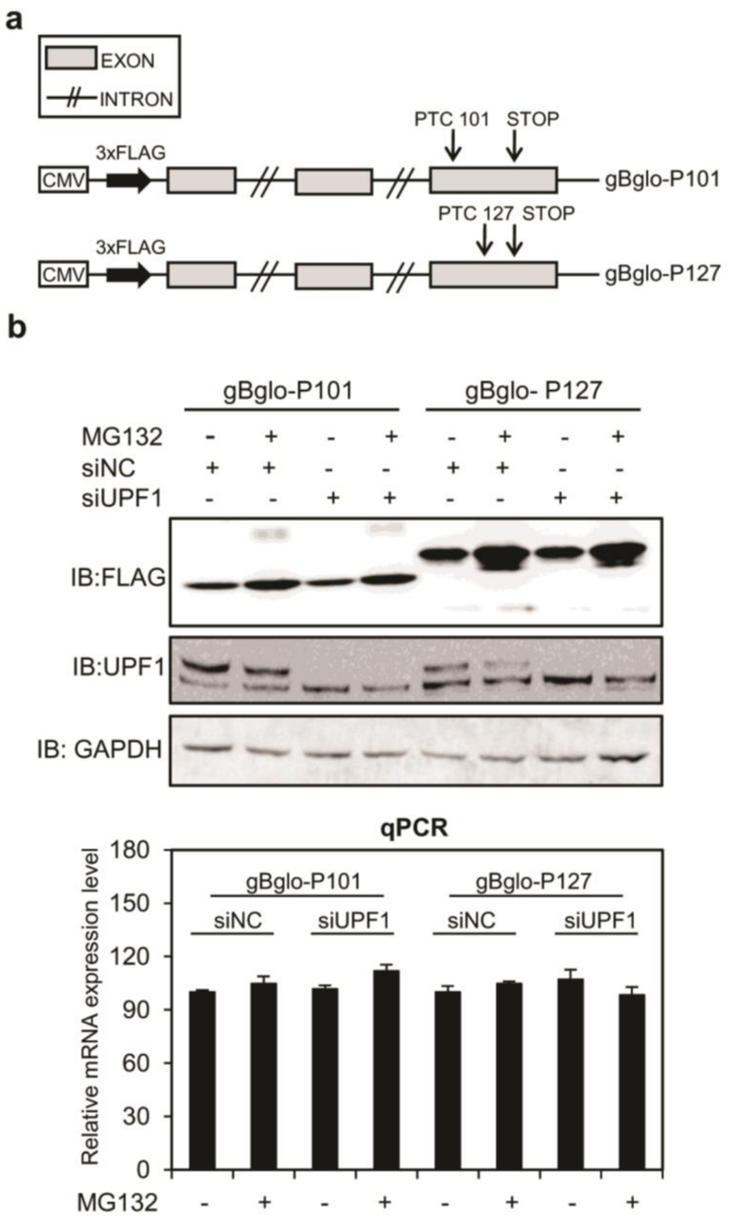

Supplementary Figure S3. Expression of protein from β-globin constructs generating mutant NMD-irrelevant mRNAs was minimally affected by UPF1 knockdown. **(a)** Schematic diagram of expression vectors containing wild-type human genomic β-globin DNA mutagenized to introduce premature termination codons (PTCs) at the indicated positions, generating NMD (nonsense-mediated decay)-irrelevant mRNAs. **(b)** Western analysis (upper) and qPCR analysis of mRNA level (lower) of HeLa cells transfected with constructs shown in (A) in the presence or absence of siRNA targeting UPF1 (siUPF1) or control siRNA (siNC), and in the presence and absence of MG132.

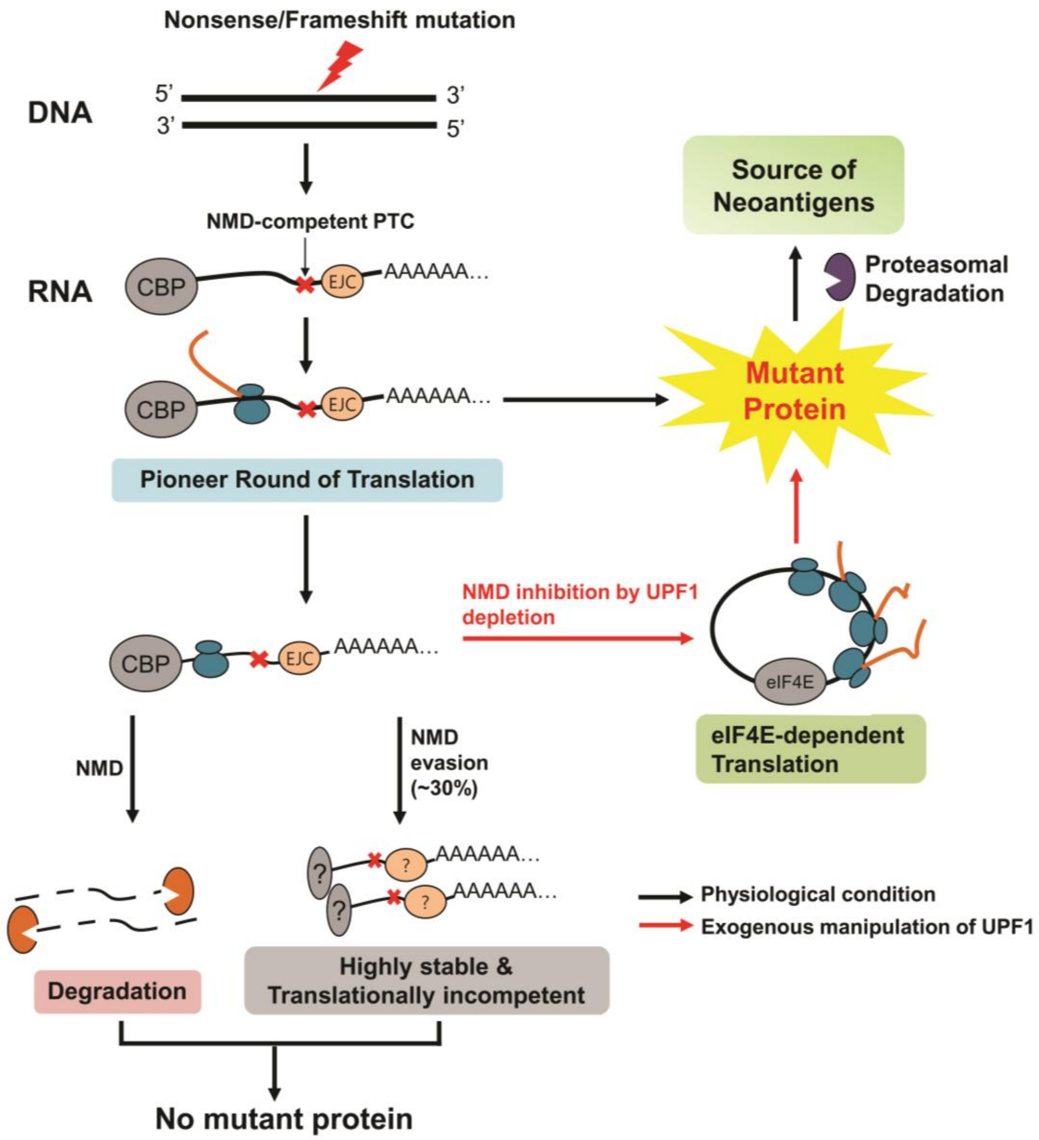

Supplementary Figure S4. Schematic model for generation of mutant proteins from PTC-containing mRNAs. Model shows the differential fates of PTC-containing mRNAs.

Supplementary table S1. Primers used for cloning and targeted nucleotide sequences of siRNAs

Gene	Direction	Sequence
PCMV10 3XFLAG-β-globin (gDNA form)	Forward	5'- AGCAACCTCAAACAGACACC
	Reverse	5'- GACCTCCCACATTCCCTTTT
PCMV10 3XFLAG-β-globin (cDNA form)	Forward	5'- TGCAACCTCAAACAGACACCA
	Reverse	5'- GCAAGAAAGCGAGCTTAGTGA
PCMV-HA-4E-BP1	Forward	5'- GTGCAGCGCACAGGAGAC
	Reverse	5'- CTCCACACGATGGCTGGT
PCMV-HA-eIF4E	Forward	5'- CTAAGATGGCGACTGTCGAA
	Reverse	5'- ATTGCTTGACGCAGTCTCCT

siRNA	Sequence
siUPF1	5'- GAUGCAGUUCCGCUCCAUU d(TT)-3'
siEIF4A3	5'- GAGCAAUCAAGCAGAUCAU d(TT)-3'
siY14	5'- CGCUCUGUUGAAGGCUGGA d(TT)-3'
siSMG1	5'- GTGTATGTGCGCCAAAGTA d(TT)-3'
siUPF2	5'- CAACAGCCCUUCCAGAAUC d(TT)-3'
siMAGOH	5'- AAAGCGUGAUGGAGGAACUG d(TT)-3'

Supplementary Information (Blots)

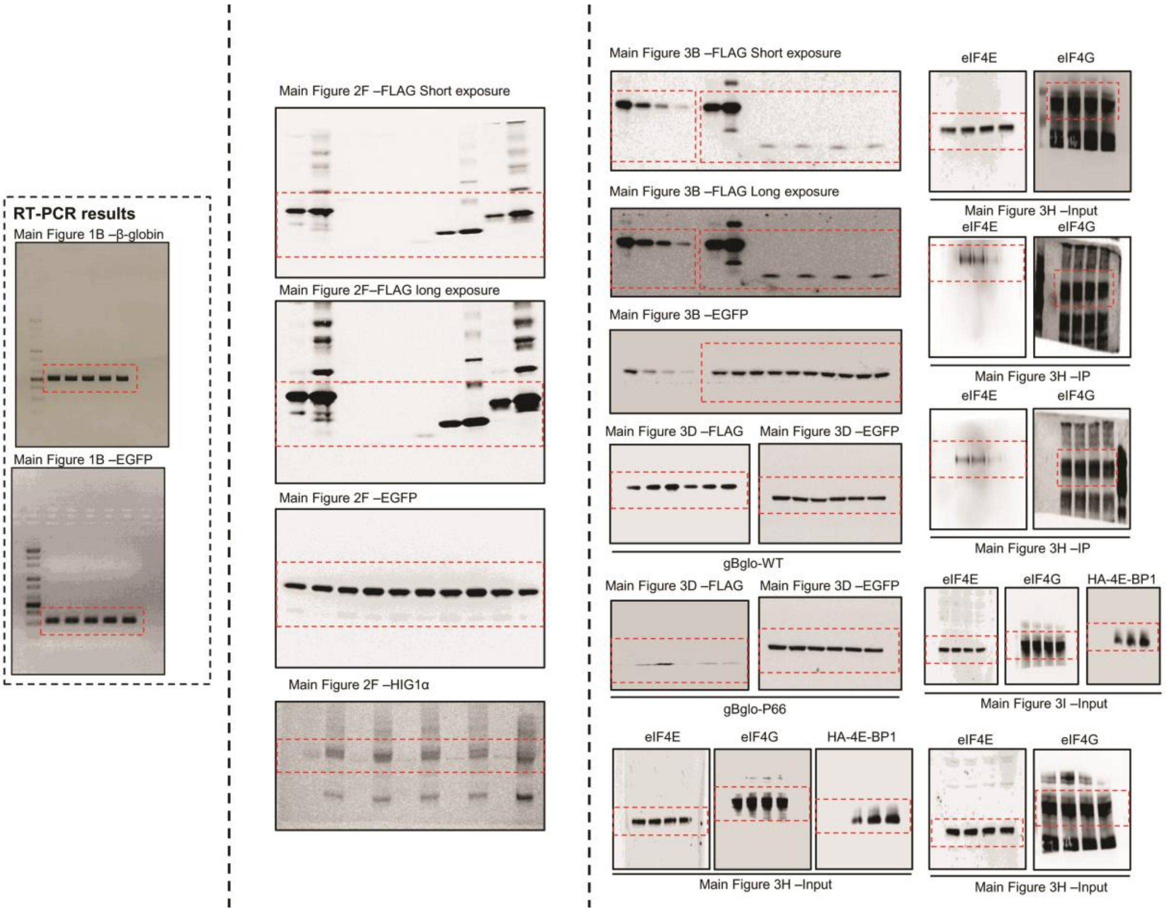

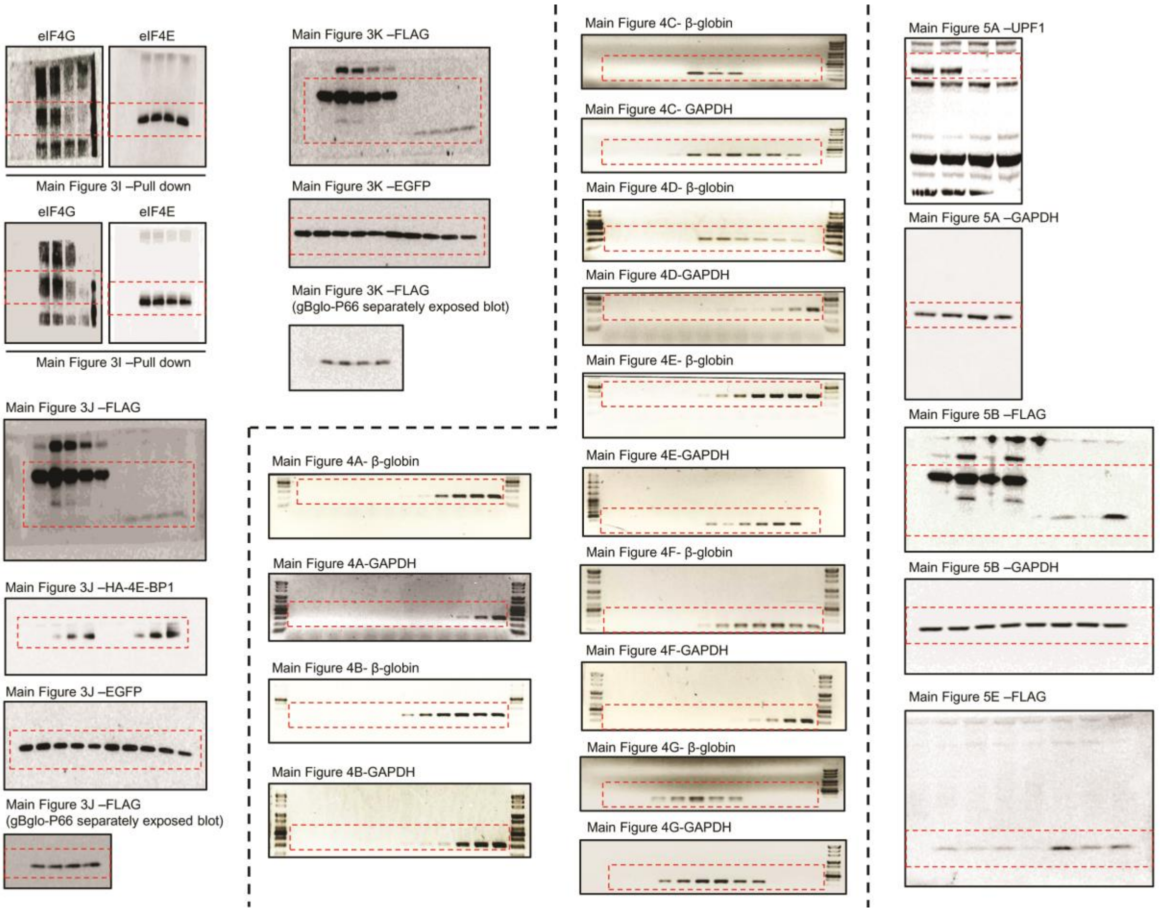
eIF4G
eIF4E
Main Figure 3I –Pull down
eIF4G
eIF4E
Main Figure 3I –Pull down
Main Figure 3J –FLAG
Main Figure 3J –HA-4E-BP1
Main Figure 3J –EGFP
Main Figure 3J –FLAG
(gBglo-P66 separately exposed blot)
Main Figure 3K –FLAG
Main Figure 3K –EGFP
Main Figure 3K –FLAG
(gBglo-P66 separately exposed blot)
Main Figure 4A- β-globin
Main Figure 4A-GAPDH
Main Figure 4B- β-globin
Main Figure 4B-GAPDH
Main Figure 4C- β-globin
Main Figure 4C- GAPDH
Main Figure 4D- β-globin
Main Figure 4D-GAPDH
Main Figure 4E- β-globin
Main Figure 4E-GAPDH
Main Figure 4F- β-globin
Main Figure 4F-GAPDH
Main Figure 4G- β-globin
Main Figure 4G-GAPDH
Main Figure 5A –UPF1
Main Figure 5A –GAPDH
Main Figure 5B –FLAG
Main Figure 5B –GAPDH
Main Figure 5E –FLAG

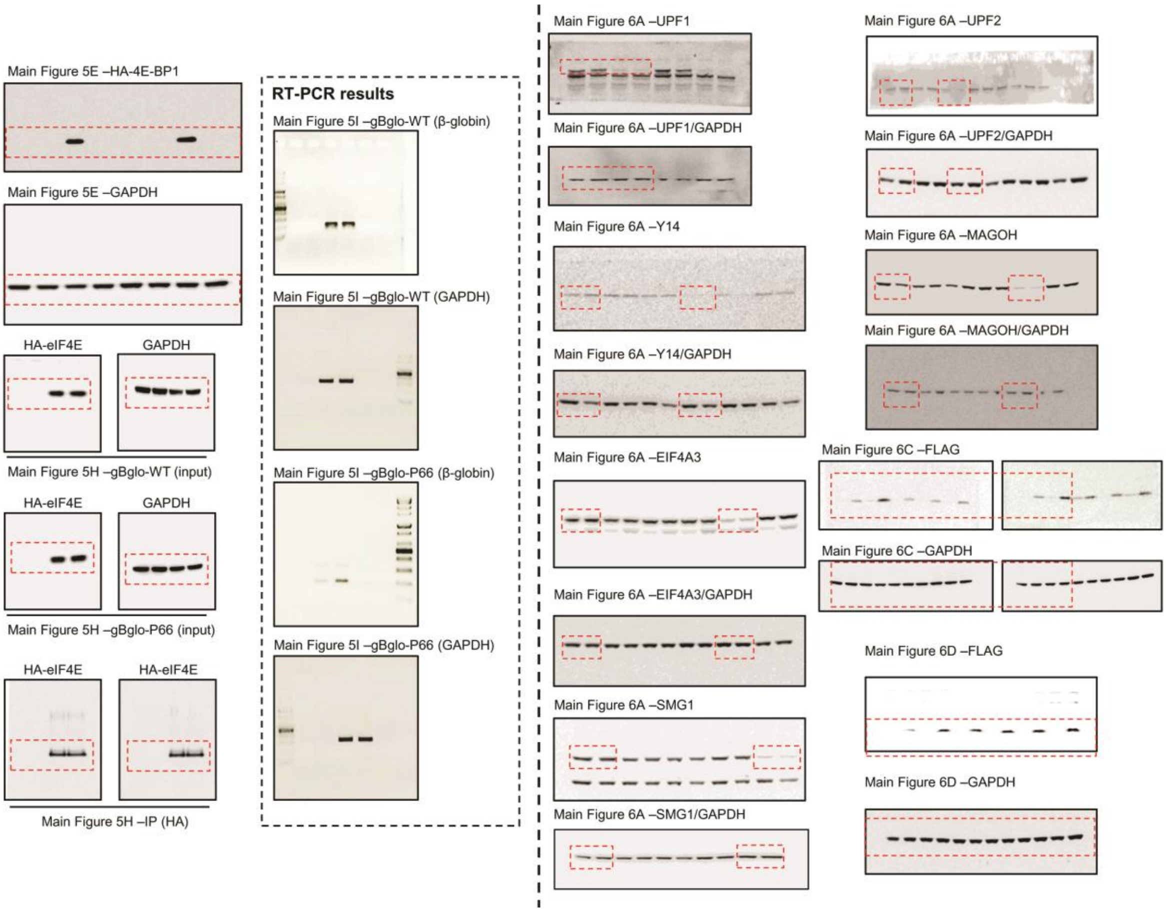
Main Figure 5E –HA-4E-BP1
Main Figure 5E –GAPDH
HA-eIF4E
GAPDH
Main Figure 5H –gBglo-WT (input)
HA-eIF4E
GAPDH
Main Figure 5H –gBglo-P66 (input)
HA-eIF4E
HA-eIF4E
Main Figure 5H –IP (HA)
RT-PCR results
Main Figure 5I –gBglo-WT (β-globin)
Main Figure 5I –gBglo-WT (GAPDH)
Main Figure 5I –gBglo-P66 (β-globin)
Main Figure 5I –gBglo-P66 (GAPDH)
Main Figure 6A –UPF1
Main Figure 6A –UPF1/GAPDH
Main Figure 6A –Y14
Main Figure 6A –Y14/GAPDH
Main Figure 6A –EIF4A3
Main Figure 6A –EIF4A3/GAPDH
Main Figure 6A –SMG1
Main Figure 6A –SMG1/GAPDH
Main Figure 6A –UPF2
Main Figure 6A –UPF2/GAPDH
Main Figure 6A –MAGOH
Main Figure 6A –MAGOH/GAPDH
Main Figure 6C –FLAG
Main Figure 6C –GAPDH
Main Figure 6D –FLAG
Main Figure 6D –GAPDH
